# Psychological quality of life and its association with academic employability skills among newly-registered students from three European faculties

**DOI:** 10.1186/1471-244X-11-63

**Published:** 2011-04-18

**Authors:** Michèle Baumann, Ion Ionescu, Nearkasen Chau

**Affiliations:** 1 INSIDE research unit, University of Luxembourg, Walferdange L-7201 Luxembourg; 2 Department of Social Work, University Alexandru Ioan Cuza, Iasi, R-700506 Romania; 3 INSERM, Univ Paris-Sud U669, Univ Paris V UMR-S0669, Paris F-75014 France

**Keywords:** students, WHOQoL-BREF, QoL-psychological, employability, academic skills, QoL-environmental, QoL-social relationships

## Abstract

**Background:**

In accord with new European university reforms initiated by the Bologna Process, our objectives were to assess psychological quality of life (QoL) and to analyse its associations with academic employability skills (AES) among students from the Faculty of Language, Literature, Humanities, Arts and Education, Walferdange Luxembourg (F1, mostly vocational/applied courses); the Faculty of Social and Human Sciences, Liege, Belgium (F2, mainly general courses); and the Faculty of Social Work, Iasi, Romania (F3, mainly vocational/professional courses).

**Method:**

Students who redoubled or who had studied at other universities were excluded. 355 newly-registered first-year students (145 from F1, 125 from F2, and 85 from F3) were invited to complete an online questionnaire (in French, German, English or Romanian) covering socioeconomic data, the AES scale and the QoL-psychological, QoL-social relationships and QoL-environment subscales as measured with the World Health Organisation Quality of Life short-form (WHOQoL-BREF) questionnaire. Analyses included multiple regressions with interactions.

**Results:**

QoL-psychological, QoL-social relationships and QoL-environment' scores were highest in F1 (Luxembourg), and the QoL-psychological score in F2 (Belgium) was the lower. AES score was higher in F1 than in F3 (Romania). A positive link was found between QoL-psychological and AES for F1 (correlation coefficient 0.29, p < 0.01) and F3 (correlation coefficient 0.30, p < 0.05), but the association was negative for F2 (correlation coefficient -0.25, p < 0.01). QoL-psychological correlated positively with QoL-social relationships (regression coefficient 0.31, p < 0.001) and QoL-environment (regression coefficient 0.35, p < 0.001).

**Conclusions:**

Psychological quality of life is associated with acquisition of skills that increase employability from the faculties offering vocational/applied/professional courses in Luxembourg and Romania, but not their academically orientated Belgian counterparts. In the context of developing a European Higher Educational Area, these measurements are major indicators that can be used as a guide to promoting programs geared towards counseling, improvement of the social environment, and services to assist with university work and facilitate achievement of future professional projects.

## Background

There are 16 million university students in Europe, with an annual growth rate of over 2% [1]. Data from the '*Organisation de coopération et de développement économiques' *(OCDE) show that 31% of students quit university dramatically without a diploma, i.e. having failed to achieve their educational/professional projects [2]. This occurs during the early university years, when young people are establishing, testing and adjusting new psychological identities [3]. However, according to the Organisation Economic Cooperation and Development's (OECD) data the proportion of university students who do not take their first diploma varies widely between countries: 40% in the United States, Mexico, New Zealand and Sweden, under 25% in Belgium, Korea, Denmark, Spain, France and Japan [2].

University students are exposed to mental difficulties related to young adulthood and have also to face with mental and social issues related to students' life [4]. It is well known that young adults experience psychological disorders, depression, unhealthy behaviours, and suicidal ideation due to their social situation and living conditions [5,6]. As students, many of them also face changes in living conditions, lifestyle and environment [7], while dealing with issues around financial support, social interaction, parental status and loneliness [8]. Many students live far from home, making them vulnerable to starting smoking and excessive alcohol consumption [9]. Today, students are expected to be competitive, adding to the pressure and leading to much more stress [10]. These issues may cause insomnia, anxiety, depression, dietary disorders and behavioural consequences [11,12] with an impact on academic achievement [13,14]. They may also generate coping strategies [15] leading to somatic disorders and violent behaviour [16]. These concerns call for a better integration of education and health care [17]. One Swedish study found that students appear to have a QoL that is lower than that of young workers of the same age [18] and is associated with academic failure, job difficulties, and diverse social outcomes later in life [19]. There is now a pressing need to explore the implications of social relationships, environment and academic employability skills for psychological issues among newly-registered students. Ultimately, interventions should be designed and evaluated, and then the most promising interventions should be implemented on a large scale.

Because social, environmental and economic contexts may vary greatly between countries it is of interest to assess whether those issues differ between universities. Three sites are considered here: in Luxemburg, Belgium and Romania. Although the Grand-duchy of Luxembourg is the smallest country in Europe (506.000 people), it is a multicultural and a multi-language society (about 170 nationalities), and the University of Luxembourg is the youngest in Europe (created in 2003). A cross-university survey "The Students' Quality of Life and Employability Skills (SQALES)", initiated by the research unit INSIDE [20] was presented to the network of the Foundation of European Regions for Research in Education and Training [21]. Two European public institutions expressed an interest in similar research; one in Liege (Belgium) and the other in Iasi (Romania). The aims of the SQALES project are: 1) to meet the recommendations adopted at Bergen (2005), under the Bologna Process, that call on universities for high levels of competition and production; and 2) to help all those involved in university education to produce guidance and advice taking some account of existing facilities (health promotion activities, employability workshops, counseling services, support for university work, student union welfare officers) and opportunities for further development [22]. In a similar vein, the Human Resources Development Canada Department of Research has created a self-rated questionnaire for university students to gauge how convinced they are of having the academic skills needed for employment according to the following dimensions: communication, interpersonal relationships, and capacity for innovation [23].

Investigating university students in Eastern and Western Europe is of interest because of disparities in health and socioeconomic issues. Compared to their Western European counterparts, Eastern Europeans have a lower prevalence of depression, weaker social support and poorer health [24]. A disproportionate number of central and eastern European students exhibit low levels of satisfaction with life, and they are more likely to believe that chance plays a major role in life achievement [25]. Psychological health, its determinants and comparisons between European countries are receiving increased attention [26,27]. Recognising psychological QoL as an issue and monitoring its fluctuation is an essential precondition to providing appropriate services and assistance to support students at university. It is agreed that a student who has a sense of well-being is more likely to achieve his/her goals, complete training, and enter the world of work. This is a major ethical issue, because we are responsible for our young people whom we ask to take up the socio-economic challenges of the current crisis. Therefore, activities with beneficial effects on mental health and well-being promote effective learning [28]. In this perspective, there is a real need for universities to evaluate the psychological difficulties of students.

Past research has shown that the WHOQoL-BREF is a relevant, reliable and valid cross-cultural scale appropriately used to measure the following four domains: physical, psychological, social relationships and environment [26]. It is the short-form of the World Health Organisation Quality of Life questionnaire. The World Health Organisation defines Quality of Life (QoL) as "the individual's perception of his/her position in life in the context of the culture and value systems in which he/she lives and in relation to his/her goals, expectations, standards, and concerns" [26]. The cross-cultural nature of the WHOQoL-BREF should make it appropriate as a means of measuring psychological status, social relationships and environment. In the European Union, QoL is considered a high social and public health policy priority that reflects wider public concerns [27]. In 2008, the European Pact signed in Brussels recognised that the mental health and well-being of the population play essential roles in the economic and social success of the Union [29]. Studies have confirmed the reliability and validity of the WHOQoL-BREF among students in various countries [30-34]. One investigation showed that a health enhancement program improved the psychological domain among medical students in Australia [30]. A high correlation was found between the psychological domain and existential well-being among social sciences students in Brazil [35]. QoL is associated with loneliness among students of health services in Turkey [36]. The psychological domain was found to be related to pparental rearing among university students in Brazil [37]. Few studies have examined the links between QoL globally and in its four domains [38]. We did not focus here on the physical domain.

The objectives of this study were to assess the QoL-psychological and to analyse its association with academic employability skills (AES), and other relevant factors among students at three European faculties: the Faculty of Language and Literature, Humanities, Arts and Education (F1), Walferdange, Luxembourg, which offers mainly vocational/applied courses; the Faculty of Social and Human Sciences (F2), Liege, Belgium, which offers mainly general/academic courses; and the Faculty of Social Work (F3), Iasi, Romania, offering mainly vocational/professional courses. Additionally, we addressed the question of whether there is a difference between students in vocational/applied and general/liberal courses/studies.

## Methods

### Population

A total of 355 first year newly-registered students in social sciences were invited to participate in the survey: 145 from Luxembourg (F1), 125 from Belgium (F2), and 85 from Romania (F3). Students who redoubled or who had studied at other universities were excluded.

### Ethics

The ethical research committees of each faculty approved the study protocol in advance, and informed consent was obtained from all respondents. Representatives of students' associations and a member of the research team provided information about the goals of the survey. The Study Directors considered the online questionnaire more appropriate than "asking students to fill in the survey during courses". This is the usual procedure in student research, but its value is open to question, particularly if the objective is to systematically collect data free of potential bias due to interaction between university staff and students.

### Procedure

Four months after the beginning of the academic year, the research team (with the cooperation of representatives of students' associations) presented information about the study and its aims and the students were assured that refusal to participate in the survey would have no effect on their academic standing. Students were asked to complete an online self-reported questionnaire via an anonymous email address in the language of their choice.

### Data collected

Three groups of variables were collected:

*a) The dependent variable QoL-psychological *was measured using the WHOQOL-BREF questionnaire (QoL-physical was not considered in this survey) [26]. The psychological subscale consists of six items on a five-point scale: (a) Bodily image and appearance, (b) Negative feelings, (c) Positive feelings, (d) Self-esteem, (e) Spirituality / Religion / Personal beliefs, and (f) Thinking, learning, memory and concentration. It has been validated in the languages used for the investigation: German [39], French [40], English and Romanian [41]. The scale is easy to administer, to complete and to score (the higher the score, the higher the perceived QoL-psychological). The internal consistency (Cronbach's alpha) coefficient was 0.77 among all students and similar to that of other studies [34,42].

*b) The variable Academic Employability Skills (AES) *was assessed using six items measuring students' perceptions of their competencies or capacities to write, think critically, solve problems, work effectively with others, lead others, and use new technology. Each student was invited to estimate his or her level on a Likert scale of 1 (not very good) to 4 (excellent). The Cronbach's alpha coefficient was 0.76 among all students. The higher the score, the higher the perceived acquisition of AES. English and French versions are available [23]. The German and Romanian versions were translated and back-translated by a team comprising a language-expert specialist in public health and a professor specialist in employability.

c) *Other factors*

- *The importance of going to university *was assessed on a four-point scale from "very important", "important", "little important" and "not important" step. The Cronbach's alpha coefficient was 0.52. The higher the score, the higher the perceived importance.

- The *QoL-social relationships and QoL-environment domains *were measured with the subscales of the WHOQOL-BREF [26]. QoL-social relationships included three five-point scale items: (a) Personal relationships, (b) Social support and (c) Sexual activity). QoL-environment included eight five-point scale items: (a) Financial resources, (b) Freedom, physical safety and security, (c) Health and social care: accessibility and quality, (d) Home environment, (e) Opportunities for acquiring new information and skills, (f) Participation in and opportunities for recreation / leisure activities, (g) Physical environment (pollution / noise / traffic / climate) and (h) Transport. They were studied as potential factors related to the QoL-psychological. Their Cronbach's alpha coefficients were 0.64 and 0.77 respectively among all students and were similar to those of other studies [34,42]. The higher the score, the higher the perceived QoL.

- *The socio-demographic characteristics: *age, sex (female/male), level of education of the father and the mother (1: End of compulsory studies; 2: Sixth form college diploma NVQ equivalent; 3: 12th grade diploma or 'A' level in Technical or professional studies; 4: University, college (higher education) diploma BTEC HND equivalent or first degree; 5: University, or specialised school diploma (higher education) Master or Doctorate / Engineer equivalent) and their professional status (manual worker / employee / senior officer).

### Statistical analysis

First, the three groups of respondent students were compared for each variable using Chi-square tests and Student's t-tests. Then multiple regression models were used in two steps: (1) we performed the regression model for the WHOQOL-BREF-psychological subscale in terms of socio-demographic characteristics and the faculty, including their interaction terms by retaining only the variables/interaction terms with p < 0.10; (2) we added to the previous model AES, QoL-social relationships, QoL-environment and their interaction terms also by retaining only the variables/interaction terms with p < 0.10.

## Results

Among the 355 newly-registered students contacted, 236 (66%) participated: 85 from Luxembourg (participation rate 55%), 82 from Belgium (participation rate 66%) and 69 from Romania (participation rate 81%).

The socio-demographic characteristics of the subjects are shown in Table 1. Samples were predominantly female: 75.3% of F1, 67.1% of F2, and 89.9% of F3. F2 was the youngest sample (mean age (SD) = 18.5 (0.87) years), compared with F3 (mean age (SD) = 19.1 (0.37)), and F1 (mean age (SD) = 21.2 (3.31)). The F1 students entered the University of Luxembourg one year later, which explains the differences in age. The highest education level among fathers and mothers (university/higher education / BTEC HND) was in F2. Employees predominated in F1 and F2 and manual workers in F3. Going to university was 'very important' for more F3 (78.3%) than F1 (55.3%) and F2 (30.9%).

**Table 1:** Socio-demographic characteristics of respondents: mean (SD) or %

		F1 (Luxembourg) N = 85	F2 (Belgium) N = 82	F3 (Romania) N = 69	p-value
Age		21.2 (3.31)	18.5 (0.87)	19.1 (0.37)	0.000
Sex	Female	75.3	67.1	89.9	0.004
	Male	24.7	32.9	10.1	
Father's educational level	1	15.9	10.9	23.2	0.000
	2	34.1	14.1	42.0	
	3	19.5	17.2	21.7	
	4	14.6	15.6	8.7	
	5	15.9	42.2	4.3	
Mother's Educational level	1	17.1	10.3	26.1	0.000
	2	25.6	5.9	33.3	
	3	28.0	22.1	26.1	
	4	17.1	42.6	5.8	
	5	12.2	19.1	8.7	
Father's professional status	Manual worker	19.5	15.0	51.5	0.000
	Employee	51.2	40.0	22.1	
	Senior officer	29.3	45.0	26.5	
Mother's professional status	Manual worker	17.1	5.1	49.3	0.000
	Employee	62.2	69.6	33.3	
	Senior officer	20.7	25.3	17.4	
Importance of going to university	Little and not important	4.7	7.4	1.4	0.000
	Important	40.0	61.7	20.3	
	Very important	55.3	30.9	78.3	

*p: Significance level of Chi square test (comparison with F1)*.

*^§ ^Level of education: 1: End of compulsory studies; 2: Sixth form college diploma NVQ equivalent; 3: 12th grade diploma or A' level in Technical or professional studies; 4: University, college (higher education) diploma BTEC HND equivalent or first degree; 5: University, or specialised school diploma (higher education) Master or Doctorate / Engineer equivalent*.

As shown in Table 2, we found that QoL-psychological, AES, QoL-social relationships and QoL-environment differed significantly between the three faculties. All scores were higher in F1: psychological (74.4 *vs*. 63.7 in F2), social relationships (75.3 *vs*. 65.2 in F3), and environment (71.7 *vs*. 56.3 in F3). The same was true for the AES (77.7 *vs*. 68.2 in F2).

**Table 2:** Comparison of QoL-psychological, academic employability skills (AES), QoL-social relationships and QoL-environment between the faculties: sex-age adjusted means (SE)

	F1 (Luxembourg) N = 85	F2 (Romania) N = 82	F3 (Belgium) N = 69	p-value
QoL-psychological	74.4 (2.0)	63.7 (1.9)	64.8 (2.1)	0.000
QoL-social relationships	75.3 (2.6)	69.0 (2.5)	65.2 (2.8)	0.020
QoL-environment	71.7 (1.8)	68.6 (1.7)	56.3 (2.0)	0.000
Academic employability skills (AES)	77.9 (1.5)	68.0 (1.5)	71.4 (1.6)	0.000

*p = Significance level of type III test*.

Table 3 shows the relationships between WHOQOL-BREF psychological subscale and socio-demographic characteristics, AES, QoL-social relationships and QoL-environment, for each faculty separately. QoL-psychological was not significantly linked with any socio-demographic factor other than the father's educational level in F3. For all faculties, QoL-psychological correlated positively with QoL-social relationships and QoL-environment. QoL-psychological correlated positively with the AES for vocational/applied courses at the faculties F1 (correlation coefficient 0.288, p = 0.009) and F3 (correlation coefficient 0.297, p = .015), but the link was negative for general/academic courses at F2 (correlation coefficient -0.255, p = 0.03). These differences concerned all items of the AES, but principally the three items problem solving, team working, and supervision/direction of others, which were associated with QoL-psychological.

**Table 3:** Relationships between QoL-psychological and socio-demographic characteristics, academic employability skills, QoL-social relationships and QoL-environment, for each faculty separately: Mean (SE) or Correlation coefficient

		WHOQoL-BREF-psychological [0-100]					
		**F1 (Luxembourg)**		**F2 (Belgium)**		**F3 (Romania)**	
	
		**Mean (SE)**	**p-value**^**1**^	**Mean (SE)**	**p-value**^**1**^	**Mean (SE)**	**p-value**^**1**^

Sex	Female	74.6 (1.9)	.962	64.3 (2.0)	.743	65.8 (2.2)	.490
	Male	74.7 (3.3)		63.2 (2.8)		61.0 (6.6)	
Father's educational level^2^	1	78.3 (4.7)	.263	52.3 (5.6)	.125	67.9 (5.1)	.045
	2	74.3 (3.0)		70.3 (5.5)		65.3 (4.5)	
	3	75.8 (3.8)		66.4 (4.7)		55.3 (4.8)	
	4	78.1 (4.3)		57.0 (4.7)		71.8 (6.9)	
	5	66.7 (4.2)		63.2 (2.9)		44.3 (10.3)	
Mother's educational level^2^	1	80.0 (4.0)	.091	56.0 (5.6)	.540	67.1 (5.2)	.451
	2	72.6 (3.4)		61.5 (8.0)		64.5 (4.9)	
	3	78.2 (3.2)		66.4 (4.4)		58.4 (4.8)	
	4	71.3 (3.9)		64.8 (2.8)		73.3 (8.9)	
	5	65.5 (4.8)		60.0 (4.2)		59.8 (7.5)	
Father's professional status	Manual worker	79.0 (3.9)	.236	62.2 (4.6)	.932	64.8 (4.2)	.686
	Employee	75.1 (2.5)		63.6 (2.7)		64.1 (5.2)	
	Senior officer	70.9 (3.2)		64.2 (2.7)		60.5 (5.0)	
Mother's professional status	Manual worker	74.4 (4.3)	.488	57.6 (8.2)	.520	63.1 (4.1)	.309
	Employee	75.8 (2.3)		63.1 (2.0)		66.6 (4.4)	
	Senior officer	70.8 (3.8)		66.5 (3.4)		57.0 (5.8)	
Importance of going to university	Little and not important	70.7 (7.3)	.192	55.9 (6.1)	.381	64.6 (17.9)	.734
	Important	71.6 (2.7)		64.7 (2.1)		60.5 (5.2)	
	Very important	77.5 (2.4)		64.6 (3.2)		64.7 (4.2)	

		correlation coefficient^3^		correlation coefficient^3^		correlation coefficient^3^	

Age		.023	.839	-.204	.075	.068	.576
QoL-social relationships		.595	.000	.458	.000	.629	.000
QoL-environment		.452	.000	.557	.000	.526	.000
Academic Employability Skills (AES - 6 items)		.288	.009	- .255	.030	.297	.015
1- Drafting/writing		.084	.456	- .009	.940	.022	.859
2- Critical spirit/having sound judgment		.103	.361	- .027	.820	.158	.201
3- Problem solving		.303	.006	- .295	.011	.352	.004
4- Team working		.316	.004	- .290	.013	.367	.002
5 - Supervision/direction of others		.214	.055	- .166	.160	.309	.011
6 - Using new technologies		.121	.281	- .116	.330	.028	.819

*^1 ^Bivariate test adjusted for sex and age*.

^2 ^*Level of education: 1: End of compulsory studies; 2: Sixth form college diploma NVQ equivalent; 3: 12th grade diploma or A' level in Technical or professional studies; 4: University, college (higher education) diploma BTEC HND equivalent or first degree; 5: University, or specialised school diploma (higher education) Master or Doctorate / Engineer equivalent*.

^3 ^*For quantitative variables*.

Table 4 shows the results obtained with the multiple regression model for WHOQOL-BREF-psychological subscale in terms of socio-demographic factors, AES, QoL-social relationships and QoL-environment subscales. No socio-demographic factor had a significant effect at the 10% level, whether or not the model included interaction terms with the faculties. There was a highly significant effect for the faculty, as QoL-psychological was higher for F1 and F3 than for F2 (regression coefficient -5.03 vs. about 2.5 for F1 and F3). More specifically, the AES related positively to QoL-psychological for F1 and F3, but negatively for F2. QoL-Psychological correlated positively with QoL-social relationships (regression coefficient 0.31, p < 0.001) and QoL-environmental (regression coefficient 0.35, p < 0.001).

**Table 4:** Relationships between QoL-psychological and Academic employability skills, QoL-social relationships and QoL-environment (multiple regression)

		WHOQoL-BREF-psychological [0-100]			
		**Regression coefficient**	**SE**	**95% CI**	**p-value**

Intercept		67.7	0.8	66.1-69.3	< 0.001
Faculty	F1 (Luxembourg)	2.37	1.19	0.03-4.71	< 0.001
	F2 (Belgium)	-5.03	1.17	-7.34--2.73	
	F3 (Romania)	2.67	1.25	0.21-5.12	
Academic employability skills		0.06	0.07	-0.07-0.19	0.377
Employability skills × faculty	F1	0.09	0.09	-0.09-0.27	0.061
	F2	-0.23	0.10	-0.43--0.04	
	F3	0.14	0.09	-0.03-0.32	
QoL-environment		0.35	0.06	0.23-0.46	< 0.001
QoL-social relationships		0.31	0.04	0.23-0.40	< 0.001

*The socio-demographic factors are not shown because p > 0.10*.

## Discussion

This study improves our understanding of the associations between the psychological quality of life, the social and environmental contexts, and the acquisition of academic employability skills among social sciences students. QoL-psychological, academic employability skills, QoL-social relationships and QoL-environment (living conditions and lifestyles on campus) differed markedly between the students from the faculties in Luxembourg (F1), Belgium (F2) and Romania (F3). QoL-psychological was positively and similarly associated with QoL-social relationships and QoL-environmental for the three faculties, and was related to the perceived importance of going to university and socio-demographic factors (professional status, level of education of the parents - all of which are major factors in creating social inequalities in health [43]) at all faculties. The association between QoL-psychological and academic employability skills (AES) was positive and similar for the faculties in Luxembourg and Romania, and opposite for the faculty in Belgium, suggesting possible variations across faculties.

Firt, it should be noted that the students' age differed between the three faculties (youngest in Belgium and lodest in Luxembourg), and that students entered university one year younger in Belgium than in Luxembourg and Romania. The over-representation of women in social sciences is well known [35,44]. In Luxembourg, it has developed a policy of Life Long Learning among the population, in particular since the opening of the university in 2003.

The literature shows that QoL-psychological, QoL-social relationships and QoL-environment differ greatly between countries (Table 5). Globally, in the three faculties we studied, the students' QoL-psychological was higher than, or similar to, that reported in other studies among university students. Students from F1 had a higher psychological QoL than did social sciences students in Brazil (74.4 vs 70.4) [35]. Students from F2 and F3 had intermediate values (respectively 63.7 and 64.8) close to that of Australian students (65.3) [32]. With regard to QoL-social relationships, the students from F1 and F2 had higher values than did students in Brazil (71.3) [35,44]. For QoL-environment, the students from F3 had a markedly lower value (56.3) than those from F1 and F2; but it was similar to that of Turkish students (52.1) [36]. This may be considered as satisfactory in the European context, given continuous changes in the demographic pattern of the student body, and difficult global economic circumstances, particularly for the Romanians [27].

**Table 5:** WHOQOL-BREF domains among students: results from the literature

Authors	Country	N	Mean age or Group age	QoL- psychological M(SD)	QoL-social relationships M(SD)	QoL- environment M(SD)
da Costa et al. [34]	Brazil	136	22.6 ± 6.13	70.4 (13.5)	71.3 (17.0)	68.4 (11.8)
Eurich et al. [43]	Brazil	67	21.2 ± 4.3	69.3 (12.3)	71.3 (15.7)	60.7 (12.7)
Hassed et al. [31]	Australia	148	18.8 ± 1.10	65.6 (16.1)	Unknown	Unknown
Kalitesi et al. [35]	Turkey	150	19	60.7 (14.7)	61.1 (14.7)	52.1 (14.3)
Li et al. [33]^1^	Thailand	407	20.5 ± 1.2	68.1 (13.7)	68.8 (15)	63.1 (12.5)
Wu and Yao [37]^1^	Taïwan	304	20.1 ± 1.7	54.4 (15)	60.6 (16.9)	58.8 (12.5)

^1 ^Scores of this study were on a 0-20 scale, we transformed them to a 0-100 scale according to the calculation of the WHOQOL Group: (score - 4)*(100/16)

Our study showed that QoL-psychological was positively linked with academic skills and knowledge related to employability among social sciences students taking vocational/professional courses in Luxembourg and Romania. We found that this association could vary across faculties as it was negative among their general/academically oriented Belgian counterparts. Two hypotheses may be raised: (1) The difference could be attributable to the two items of AES, namely problem solving and team working, which had the same link as between AES and QoL-psychological; it may suggest that the general/academically orientated training for the Belgians may include general aspects of social sciences and pay less attention to certain issues such as problem solving and team working than the other students receive with their vocational/professional orientation in Luxembourg and Romania; (2) The Belgians were younger and may be less satisfied with their AES as shown by their lower score (68.0 vs. 77.9 in Luxembourg, and 71.4 in Romania). Students with higher AES would find it insufficient to assure their professional future and this may impact on their QoL-psychological. Among the AES items, problem solving and team working may be more complex than writing and having sound judgment (which were not related to QoL-psychological at any faculty) so that younger age may impact on them. However, with recent reforms in European universities, students must, during their course, not only acquire the knowledge necessary to obtain the diploma they are preparing for, but also integrate the competences sought by employers. Our findings suggest that improving training in problem solving and team working for the general/academically orientated training would be helpful for the Belgians. Canadian research [23] highlighted some interesting points when comparing the academic employability skills of their students and post-graduates. It was observed that social sciences graduates who had worked for at least two years attained higher AES. The universities should thus provide students with high level AES and a good quality of life in the social relationships, environmental and psychological domains. The feeling of personal effectiveness is a major determinant of the process of belief necessary to work towards a potential occupation [45].

We found that, for the three faculties, the higher the psychological QoL, the higher QoL-social relationships and QoL-environment. When we compare our results with those in the literature, Luxembourg students seemed to have a comfortable QoL for all these domains. Conversely, Belgian students had values approaching those of Thai students, with a QoL-environment near to that of students in social sciences in Brazil. Romanian students had the lowest values, even lower than those of Thai students. In agreement with our results, a recent study among Turkish students showed that the better the mental health, the better the social relations, in particular romantic relationships [8]. Another recent study confirmed that the prevalence of psychiatric disorders among first-year students led to increased difficulty in adapting to university education. However, the cross-sectional study design prevents us from drawing conclusions about the direction of the link: poorly perceived adjustment to the academic environment may increase the risk of psychiatric disorders or psychiatric disorders may increase the difficulties in adjusting to the academic environment, or both may be caused by other factors not investigated here [19]. Although satisfaction with going to university was reported to be an important factor for academic achievement [25], our study failed to reveal an association between perceived importance of going to university and QoL-psychological at any of the three faculties.

This project is novel for several reasons. First, there are few investigations into QoL-psychological using the WHOQOL-BREF among students. Second, to our knowledge, no study has explored the link between psychological quality of life and academic employability skills. Third, social sciences and newly-registered students are not the populations assessed most frequently. We focused on newly-registered students because of the need to identify issues and to deal with problems as early as possible. This is important as it enables us to identify some problems and challenges related to transition periods. Every year, many students leave university without a diploma [2,46]. The beginning of university life is an important period of change for young adults in terms of interactions between individual bio-psychological characteristics and societal demands [4]. Young adulthood has great potential for personal growth or for failure that may impact on feelings of independence and control. The framework of our study responds to the new missions for universities to promote, for students, quality of life, employability and the participatory process [46].

### Some several limitations

First, the survey was conducted among newly-registered volunteers and examined only one social sciences faculty per country; therefore, the results could not be generalized. Second, although the population is small, the participation rate of 66% is rather high for a web-based survey [47]. Unfortunately, no data are available with which to assess the participation bias. Third, a web-based self-administered questionnaire might lead to bias in terms of responses to questions [48]. However, the web-format WHOQOL-BREF is considered equivalent to the paper version [47], and the quality of the completed questionnaires was very high. Fourth, an advantage of the WHOQOL-BREF is that it has been used in cross-university surveys on mental health.

## Conclusion

This study shows that QoL-psychological, academic employability skills, QoL-social relationships and QoL-environment differed considerably between newly-registered students from the social sciences faculties in Luxembourg, Belgium and Romania. They were rather high among Luxembourg students and lower among the others. At all three faculties, QoL-psychological positively correlated with QoL-social relationships and QoL-environment, but the relationships between QoL-psychological and academic employability skills varied across faculties. It was positive for Luxembourg and Romanian students following vocational/professional orientated courses and negative for younger Belgian students in general/academically orientated training. Social status of parents defined by educational level and occupation did not play a role in this association. However, the academic employability skills and the quality of life were not at their best levels (score varying from 68.0 for Romanian, 71.4 for Belgian, and 77.9 for Luxembourg students on a 0-100-point scale). Interventions can therefore be made to evaluate the difficulties and help students adapt to the university environment. In some universities, tutoring groups have been created to help students with their university work, working methods, interaction with other students, familiarity with academic requirements, stress reduction, achievement of feelings of control and autonomy and to promote appropriate coping strategies [45]. Efforts should also be devoted to improving pedagogic supervision of first-year students. Personal interviews can be used to advise on how to ensure that academic efforts correspond to professional projects and capacities [49]. Further studies are needed to explore these issues in other universities and for all undergraduate years, and especially among students who leave university without a diploma.

## Competing interests

The authors declare that they have no competing interests.

## Authors' contributions

MB conceived the protocol and tools of the survey, carried out the study and had the main responsibility for writing the manuscript.

II participated in conceiving and organising the survey.

CN participated in conceiving the statistical analyses and writing the manuscript.

The authors read and approved the final manuscript.

## Pre-publication history

The pre-publication history for this paper can be accessed here:

http://www.biomedcentral.com/1471-244X/11/63/prepub
